# Work-Related Psychosocial Factors and Mental Health Problems Associated with Musculoskeletal Pain in Nurses: A Cross-Sectional Study

**DOI:** 10.1155/2016/9361016

**Published:** 2016-11-03

**Authors:** Tiina Freimann, Mati Pääsuke, Eda Merisalu

**Affiliations:** ^1^Tartu University Hospital, Puusepa 1a, 50406 Tartu, Estonia; ^2^Institute of Family Medicine and Public Health, Faculty of Medicine, University of Tartu, Ravila 19, 50411 Tartu, Estonia; ^3^Institute of Exercise Biology, University of Tartu, Ravila 14a, 50411 Tartu, Estonia; ^4^Institute of Technology, Estonian University of Life Sciences, Kreutzwaldi 56, 51014 Tartu, Estonia

## Abstract

*Background*. Musculoskeletal pain is the most common cause of incapacity among nurses. This study aimed to report the prevalence of musculoskeletal pain among hospital nurses and to explore the associations of work-related psychosocial factors and mental health problems with musculoskeletal pain.* Methods*. A cross-sectional survey was carried out among registered nurses at Tartu University Hospital during April and May 2011. Binary logistic regression was used to assess the associations between dependent and independent variables.* Results*. Analysis was based on 404 nurses (45% of the hospital's nursing population). The overall prevalence of MSP was 70% in the past year and 64% in the past month. Lower back (57%) and neck (56%) were the body areas most commonly painful in the past year. Higher quantitative and emotional demands, work pace, low justice and respect in the workplace, influence on work organisation, and role conflicts were significantly associated with musculoskeletal pain among nurses (*p* < 0.05). All mental health problems and most strongly somatic stress symptoms were associated with musculoskeletal pain.* Conclusions*. Work-related psychosocial risk factors and mental health problems, especially somatic stress symptoms, have an important impact on the occurrence of musculoskeletal pain among university hospital nurses.

## 1. Background

According to 2014 Estonian Health Board statistics, musculoskeletal disorders account for 74% of all occupational diseases in Estonia and were the most common reason for receiving medical absence benefits [1]. Globally, nurses are one of the occupational groups with high prevalence of musculoskeletal pain (MSP) [2–10], which results in adverse health consequences for individuals, health care institutions, and society.

Epidemiologic studies have demonstrated that work-related physical and psychosocial factors (PSFs) and individual characteristics play an important role in the development of MSP [11]. These risk factors have independent or interactive effects on the development of MSP and they may affect MSP directly or indirectly as a result of individuals' stress experience [12, 13]. Risk factors may reinforce each other and or their effects may be mediated by cultural factors and health beliefs [14]. The literature review of Sherehiy et al. (2004) provides evidence that work-related PSFs, especially work organisation problems and social relationships at work, are strongly related to musculoskeletal outcomes in the nursing population [15].

Previous studies in Estonia have shown high prevalence of MSP and mental health problems (MHPs) (including stress, burnout, and somatic stress symptoms) among university hospital nurses [16, 17]. The occurrence of MSP in nurses was associated with physical work load, emotional exhaustion, and somatic stress symptoms [16]. Somatic stress symptoms were associated with several PSFs, including workload, emotional demands, social relationships, trust regarding the management, and justice, and respect in the workplace [17]. However, due to multifactorial nature of MSP, earlier studies have given controversial information about the risk factors for MSP. The aim of our study was to describe the prevalence of MSP and to explore the associations of work-related PSFs and MHPs with MSP among nurses at the university hospital in Estonia.

## 2. Methods

A cross-sectional study was carried out among registered nurses during April and May 2011 at Tartu University Hospital (TUH), Estonia. All 906 full-time registered nurses who had been employed at the hospital for at least one year were invited to complete an electronic questionnaire. Three reminders of the survey were sent to these nurses over a six-week period. In total, 409 nurses have completed the questionnaire (response rate 45%). The study was approved by the Research Ethics Committee of the University of Tartu (protocol number 202T-19) and conducted in accordance with the Helsinki Declaration. The voluntary and anonymous nature of participation was emphasized in the letter of invitation and through verbal communication.

The questions from the Nordic Musculoskeletal Questionnaire (NMQ) were used to assess the prevalence of MSP at six anatomical areas of the body (lower back, neck, shoulder, elbow, wrist/hand, and knee) lasting for longer than a day during the past year and past month [18]. Information about sociodemographic characteristics and work history was also collected. Version two of the Copenhagen Psychosocial Questionnaire (COPSOQ II) was used to evaluate work-related PSFs and MHPs among the participants [19]. Work-related PSFs were assessed using 85 items grouped into 24 scales that covered the following five psychosocial domains: demands at work; work organisation and job contents; interpersonal relationships and leadership; the work-individual interface; and values in the workplace. MHPs were measured using 24 items grouped into 6 scales: sleeping troubles, burnout, stress, depression symptoms, somatic stress symptoms, and cognitive stress symptoms.

Statistical analysis was performed using IBM SPSS Statistics for Windows Version 24.0 (IBM Corp., Armonk, NY). First, descriptive statistics were used to analyse the data. The number and percentage were calculated to describe the prevalence of MSP in the past year and past month. For the analysis of PSFs all the items were scored from 0 to 100 points (the five response options were 0, 25, 50, 75, and 100 and the four response options 0, 33.3, 66.7, and 100) to make the scoring on the different scales comparable [20]. The total score on a scale was the mean of the scores of the individual items. Binary logistic regression was used to assess the associations of work-related PSFs and MHPs with MSP and summarised by odds ratios (ORs) with 95% confidence intervals (CIs). For regression analysis, the five and four response options were used. In each analysis, the nurses who did not have the outcome under consideration were included in the referent category.

## 3. Results

The questionnaires were completed by 409 of the 906 nurses invited to take part in the study (a response rate of 45%). After checking for compliance with the criteria, five respondents were excluded because they had worked at the hospital for less than a year. A total of 404 questionnaires were used in the analysis.

Table 1 shows the demographic and lifestyle factors of the participants. Most of the nurses were women (98%) and their ages ranged from 23 to 69 with a mean age of 40. More than half of the participants (56%) had worked in their job for more than 10 years and one-fifth were employed as nursing managers. Among the participants, 51% of the nurses used analgesics for MSP more than once a month. Current smokers constituted 20% of the participants, and 25% of nurses used alcohol one or more times per month.

Table 2 presents the mean scores and 95% confidence intervals for self-reported PSFs and MHPs. The work-related PSFs with the highest mean scores were meaning of work; role clarity; expectation of having to hide emotions; social relationships at work; mutual trust between employees. The lowest mean scores were recorded for job insecurity; workload; influence on work organisation; role conflicts; and work-family conflict. Stress and burnout showed the highest mean scores for MHPs.

Seventy percent of the participants reported having at least one body area with MSP lasting for longer than a day within the past year, and 64% reported having MSP in the past month (Table 3). The lower back and neck were the sites most often affected by pain.

Table 4 presents the associations of PSFs and MHPs with MSP over the past year and past month. Work-related PSFs such as high quantitative and emotional demands, work pace, low justice and respect in the workplace, and role conflicts were significantly associated with MSP among nurses. Work-individual interface factors such as job dissatisfaction and work-family conflicts also showed positive correlation with MSP. The most significant associations were observed between somatic stress symptoms (stomach ache; headache; palpitations; tension in various muscles) and MSP.

## 4. Discussion

Our study indicated that as in many other countries [2–10] MSP is quite common among TUH nurses. The prevalence of MSP in any body area occurred in 70% of nurses over the past year, which was lower than in a previous study in Estonia (84%) [16]. There could be several reasons for this variation, but one of them could be related to the differences in study design. The smaller sample size and higher prevalence of MSP in the preliminary study could have been due to subjects with pain being more interested in participating in the first study in our university hospital. Another reason may be the time during which the data were collected. While the previous study was performed from October 2008 to February 2009 (autumn/winter), the present study was carried out during April and May 2011 (spring). The effect of season on the occurrence of MSP has received insufficient attention in scientific literature.

The prevalence of MSP varies across countries and studies. For example, very different prevalence of MSP was obtained in two Swedish studies [9, 21]. According to the research of Nilsson et al. (2010) [21], the prevalence of MSP among Swedish nurses was significantly lower compared to the results of a study by Josephson et al. (1997) [9] and with those from other countries. The decrease in MSP prevalence among nurses in Sweden may be supported by the long period between the two surveys. However, a difference in the prevalence of MSP among nurses within the same country has also been found in Japan [5, 22], where the studies were carried out at shorter intervals.

Comparing the mean scores of work-related PSFs between Estonian and US nurses [23], higher values occurred among Estonian nurses for the following factors: meaning of work; role clarity; access to the information; mutual trust between employees. Lower comparative scores for Estonian nurses occurred for workload, role conflicts, and their influence on the work organisation. In comparison with other salaried workers in Estonia [24], TUH nurses provided higher scores for work pace and emotional demands and lower scores for social relationships at work and social support from supervisors. Influence on work organisation and justice and respect in the workplace were also scored quite low (33 and 49 on a 100-point scale). A low influence on the work organisation seems to be a common problem for nurses also in the other countries, for example, in Denmark (33) and the US (46) [23, 25]. The meaning of low influence on work organisation is expressed in terms that often the nurses have no choice in deciding on the amount of work and how or what to do at work.

Justice and respect in our study was scored considerably lower than for other salaried workers in Estonia (64.9) [24]. A low level of justice and respect has not been mentioned as a risk factor in earlier studies, but it was found to be associated with the occurrence of MSP in present study. There are a number of strategies that could be used to promote justice and respect at the workplace. Based on the results of present study, it is important to ensure that the nurses' work would be shared and conflicts at the workplace would be resolved in a fair way. All suggestions from the nurses should be treated more seriously by the management and the nurses should be appreciated when they have done a good job.

High quantitative and emotional demands, work pace, influence on work organisation, and role conflicts were found to be associated with MSP among TUH nurses. These results are similar to previous studies that have been conducted among nurses [3, 10, 26, 27]. Based on these results, it would be important to monitor nurses' work pace and quantitative and emotional job demands and to analyse work roles and work organisation. Work-individual interface factors such as job dissatisfaction and work-family conflicts also played a role in the occurrence of MSP. Van der Heijden et al. (2008) examined work-home interference among nurses and found that it could mediate the effects of job demands on health [28]. The hypothesis that family-work interference factors mediate the effects of psychosocial risk factors on MSPs would require further investigation. Although all MHPs were associated with MSP, the most significant association was observed however between somatic stress symptoms (stomach ache; headache; palpitations; tension in various muscles) and MSP. This finding supports an earlier study [29], in which somatic symptoms were found to be the leading determinant of MSP. A previous international CUPID (Cultural and Psychosocial Influences on Disability) study in Estonia and other countries demonstrated also positive associations between distressing somatic symptoms and MSP among nurses [16, 29–31].

Older age and perceived poor health were important risk factors for the occurrence of MSP in TUH nurses. This is in agreement with the findings of other researchers [3, 15, 21]. Because of that, all regression analyses in this study were adjusted for age and self-rated health. Surprisingly, most of the negative lifestyle factors (smoking, alcohol use, and taking pain medicines) were not associated with MSP among TUH nurses, as they have found to be risk factors in previous studies [3, 10]. A weak association concerning MSP in the past month was found only between body mass index and length of service.

In summary, our study supports the earlier international scientific knowledge about the associations of work-related PSFs and MHPs with the occurrence of MSP in nursing profession and provides some additional information about possible effect of justice and respect on the prevalence of MSP among nurses.

Our study had some important limitations. First, despite providing repeated reminders, the response rate to requests to participate in the survey was modest. Nonetheless, the study sample was homogeneous according to average age (40 years) and working experience (56% and 54%, resp.). Second, the cross-sectional design of the study left some uncertainty about the causal relationship between dependent and independent variables. Third, the assessment of exposures was based on self-reporting. It could be that distress caused by MSP made some participants more likely to report poorer mental health, affecting their scores for PSFs.

## 5. Conclusions

This study suggests that the prevalence of MSP among hospital nurses is high. Lower back and neck are body areas most frequently affected by pain. Work-related psychosocial risk factors (quantitative and emotional demands, work pace, low justice and respect in the workplace, influence on work organisation, and role conflicts) and mental health problems, especially somatic stress symptoms, appear to have an important impact on the occurrence of musculoskeletal pain among university hospital nurses. The results of this study suggest that there would be an improvement in the PS work environment of hospital nurses.

## Figures and Tables

**Table 1:** Participants' demographic and lifestyle characteristics.

Variable	*N*	%
Age (years)		
22–29	88	21.8
30–39	116	28.7
40–49	105	26.0
50–59	95	23.5
Gender		
Female	401	98.30
Male	7	1.7
Occupation		
Nursing	323	79.9
Nursing management	81	20.1
Work tenure		
<5	80	19.8
5–10	96	23.8
>10	228	56.4
BMI		
≤24.9	207	51.2
≥25.0	197	48.8
Taking pain medicine during the past 3 months		
Never	60	14.9
Seldom	140	34.6
One to several times a month	115	28.5
One to several times a week	73	18.0
Every day	16	4.0
Smoking		
Never and ex-smoker	322	79.7
Current smoker	82	20.3
Alcohol drinking during the past 3 months		
Never	95	23.5
Seldom	176	43.6
One to several times a month	101	25.0
One to several times a week	25	6.2
Every day	7	1.7

**Table 2:** Work-related psychosocial factors and mental health problems for 404 nurses.

Psychosocial factors (scales)	Number of items	Mean	95% CI
Work demands			
Quantitative demands (workload)	4	32.2	30.5–33.9
Work pace	3	66.3	64.8–68.0
Cognitive demands	4	67.2	65.6–68.7
Emotional demands	4	57.1	55.3–58.8
Expectations of hiding emotions	3	73.3	71.6–75.1
Work organisation and job contents			
Influence on work organisation	4	33.3	31.3–35.4
Possibilities for development	4	68.6	67.1–70.2
Meaning of the work	3	80.2	78.7–81.7
Commitment to the work	4	63.7	61.7–65.6
Interpersonal relationships and leadership			
Access to the information	2	63.2	61.2–65.3
Rewards (recognition)	3	57.6	55.5–59.7
Role clarity	3	78.9	77.5–80.2
Role conflicts	4	35.9	34.0–37.8
Quality of leadership	4	59.6	57.4–61.9
Social support from colleagues	3	59.9	57.8–62.0
Social support from supervisor	3	57.8	55.2–60.5
Social relationships at work	3	71.4	69.5–73.4
Work-individual interface			
Job insecurity	4	18.4	16.4–20.3
Job satisfaction	4	65.5	64.0–66.9
Work-family conflict	4	43.5	40.9–46.0
Values in the workplace			
Mutual trust between employees	3	71.1	69.2–73.0
Trust regarding management	4	63.7	62.3–65.2
Justice and respect	4	49.3	46.8–51.9
Social inclusiveness	4	61.3	59.8–62.9
Mental health problems			
Stress	4	41.2	39.5–42.8
Somatic stress symptoms	4	30.8	29.3–32.3
Cognitive stress symptoms	4	26.6	25.0–28.1
Depression symptoms	4	30.9	29.3–32.5
Sleeping troubles	4	32.7	30.7–34.6
Burnout	4	45.1	43.4–46.7

**Table 3:** Prevalence of musculoskeletal pain in the past year and past month.

Body area with pain	Past year		Past month	
	*N*	%	*N*	%
Lower back	230	56.9	159	39.4
Neck	225	55.7	174	43.1
Shoulder	125	30.9	106	26.2
Elbow	50	12.4	40	9.9
Wrist/hand	81	20.0	65	16.1
Knee	126	31.2	93	23.0
MSP in any body part	283	70.0	257	63.6

Note. All percentages are calculated from the total sample (*N* = 404).

**Table 4:** Associations with musculoskeletal pain in past year and month.

Psychosocial factors (scales)	MSP in the past year	MSP in the past month
	OR^*∗*^ (95% CI)	OR^*∗*^ (95% CI)
Work demands		
Quantitative demands (workload)	1.13 (1.02–1.25)	1.09 (1.00–1.20)
Work pace	1.14 (0.99–1.31)	1.17 (1.04–1.32)
Cognitive demands	1.12 (1.01–1.25)	1.08 (0.99–1.19)
Emotional demands	1.10 (1.00–1.24)	1.17 (1.08–1.28)
Expectations of hiding emotions	1.06 (0.95–1.20)	1.06 (0.95–1.17)
Work organisation and job contents		
Influence on work organisation	0.88 (0.81–0.96)	0.92 (0.85–0.99)
Possibilities for development	1.01 (0.91–1.12)	1.01 (0.92–1.11)
Meaning of the work	1.02 (0.89–1.17)	1.01 (0.90–1.15)
Commitment to the work	0.96 (0.88–1.05)	0.98 (0.91–1.06)
Interpersonal relationships and leadership		
Access to the information	0.92 (0.78–1.08)	0.89 (0.77–1.03)
Rewards (recognition)	0.92 (0.82–1.03)	0.92 (0.84–1.01)
Role clarity	0.99 (0.85–1.15)	0.96 (0.85–1.10)
Role conflicts	1.09 (1.00–1.19)	1.09 (1.01–1.17)
Quality of leadership	0.94 (0.87–1.02)	0.98 (0.92–1.05)
Social support from colleagues	0.85 (0.76–0.95)	0.96 (0.88–1.06)
Social support from supervisor	0.94 (0.86–1.02)	1.01 (0.94–1.08)
Social relationships at work	0.89 (0.78–1.02)	0.90 (0.81–1.01)
Work-individual interface		
Job insecurity	1.01 (0.92–1.11)	0.97 (0.89–1.05)
Job dissatisfaction	1.19 (1.02–1.39)	1.16 (1.01–1.33)
Work-family conflict	1.14 (1.03–1.25)	1.17 (1.07–1.27)
Values in the workplace		
Lack of trust between employees	1.20 (1.05–1.38)	1.09 (0.98–1.22)
Lack of trust regarding the management	1.16 (1.05–1.28)	1.08 (0.99–1.18)
Lack of justice and respect	1.18 (1.08–1.30)	1.11 (1.03–1.19)
Low social inclusiveness	1.02 (0.92–1.12)	1.07 (0.98–1.16)
Mental health problems		
Stress	1.35 (1.20–1.53)	1.34 (1.20–1.49)
Somatic stress symptoms	1.71 (1.44–2.02)	1.51 (1.32–1.73)
Cognitive stress symptoms	1.19 (1.06–1.34)	1.14 (1.03–1.25)
Depression symptoms	1.19 (1.06–1.33)	1.23 (1.11–1.37)
Sleeping troubles	1.21 (1.09–1.34)	1.16 (1.06–1.27)
Burnout	1.23 (1.10–1.39)	1.32 (1.18–1.48)

^*∗*^Adjusted for age and self-rated health.

## Abbreviations

CI: Confidence interval
COPSOQ: Copenhagen Psychosocial Questionnaire
MHPs: Mental health problems
MSP: Musculoskeletal pain
PSFs: Psychosocial work factors
NMQ: Nordic Musculoskeletal Questionnaire
SD: Standard deviation.

## Additional Points

*Availability of Supporting Data*. The datasets generated and analysed during the current study are not publicly available to preserve the privacy of participants; however they are available from the corresponding author on reasonable request.
